# Lysyl oxidase‐like protein secreted from an acidophilic red alga, *Cyanidium caldarium*

**DOI:** 10.1002/pld3.84

**Published:** 2018-10-08

**Authors:** Tatsuya Tomo, Akinori Okumura, Takehiro Suzuki, Mirai Okuhara, Ruriko Katayama, Noboru Isayama, Ryo Nagao, Masako Iwai, Naoshi Dohmae, Isao Enami

**Affiliations:** ^1^ Department of Biology Tokyo University of Science Tokyo Japan; ^2^ Graduate School of Science Tokyo University of Science Tokyo Japan; ^3^ Department of Integrated Sciences in Physics and Biology College of Humanities and Sciences Nihon University Tokyo Japan; ^4^ Biomolecular Characterization Unit RIKEN Center for Sustainable Resource Science Saitama Japan; ^5^ Research Institute for Interdisciplinary Science Okayama University Okayama Japan; ^6^ School of Life Science and Technology Tokyo Institute of Technology Yokohama Japan

**Keywords:** cell division, *Cyanidium*, endospore, eukaryote, lysyl oxidase

## Abstract

*Cyanidium caldarium* is a primitive acidophilic red alga which grown optimally at pH 1–3. When the alga was cultured at pH 6, which is the upper limit of acidity for its survival, most of the algal cells became large cells with four endospores which did not split into daughter cells. This suggests that the alga survives in the endospore state at pH 6 to protect against nutrient uptake deficiency due to low pH gradient across the cell membranes. The alga was also found to secrete an extracellular protein specifically at pH 6. The protein was identified to be lysyl oxidase‐like protein, which had been reported to be widely distributed in the animal kingdom but not yet found in the plant kingdom. In the plant kingdom, only two primitive acidophilic algae, *C. caldarium* and *Cyanidioschyzon merolae*, possess a gene encoding this protein.

## 1 INTRODUCTION

The transition from prokaryotic cells into eukaryotic cells is important in biological evolution. The order Cyanidiales that consist of three genera *Cyanidioschyzon*,* Cyanidium* and *Galdieria* are the only photosynthetic organisms which have existed for hundreds of millions of years in acidic hot springs (Reeb & Bhattacharya, 2010). Since the common characteristics of these species are shared with the prokaryotic cyanobacteria and with the eukaryotic Rhodophyta (red algae), this makes them good candidates for covering the evolutionary gap between the prokaryotic and eukaryotic cell (Seckbach, 1987). These species show progressive morphological and biochemical steps from primitive cyanobacterial features of *Cyanidioschyzon* to *Cyanidium* and to further advanced *Galdieria* (Seckbach, 1995). The first alga, *Cyanidioschyzon merolae*, resembles the cyanobacteria in its mode of division by binary fission and simple eukaryotic cellular features, and it has the smallest of nuclear and chloroplast DNA in eukaryotic algae (Matsuzaki et al., 2004). The second member, *Cyanidium caldarium,* indicates an intermediate status in cyanidiaceaen line. The third alga, *Galdieria sulphuraria* which shows advanced features, has more developed organelles, a multilobed chloroplasts, and storage glucans closer to the lower *Rhodophyta* (Seckbach, 1995).

*Cyanidium caldarium* strain RK‐1 used in this study is a primitive unicellular red alga which can grow at pH values as low as 0 (Allen, 1959), and the pH optimum for growth is between 1.0 and 3.0 (Allen, 1959; Doemel & Brock, 1971). The alga is an obligate autotroph and has a simple cellular structure, with each cell having one nucleus, one mitochondrion, and a large single chloroplast but no vacuoles (Enami, Nagashima, & Fukuda, 1975). The algal cells multiply by internal divisions forming four endospores within a mother cell (Ford, 1984). It has a thick cell wall which is largely (50%–55%) proteinaceous, unlike most of the red algae (Bailey & Staehelin, 1968). Analysis of plastid gene clusters indicated that *Cyanidium* and *Cyanidioschyzon* were closely related and that they formed a sister group distinct from other rhodophytes (Ohta et al., 1997).

Despite the extremely acidic environment, the intracellular pH of *Cyanidium* cells has been indicated to be neutral (Enami & Kura‐Hotta, 1984; Kura‐Hotta & Enami, 1981, 1984). The intracellular pH of *Cyanidium* cells was directly measured as a function of external pH by ^31^P‐NMR (Enami, Akutsu, & Kyogoku, 1986). Cells incubated under aerobic conditions in the dark or anaerobic conditions in the light were found to maintain their intracellular pH within a narrow range (pH 6.8–7.0) even when the external pH was changed from pH 1.2 to 8.4 (Enami et al., 1986). In contrast, the intracellular pH of cells incubated under anaerobic conditions in the dark was acidified as the external pH decreased (Enami et al., 1986). These indicate that when ATP production by either respiration or photosynthesis is suppressed, passive transport of protons into the cells from acidic external medium results in an intracellular acidification. If ATP production is functional, cells are able to pump out protons and maintain their intracellular pH at a constant, physiological range. On the basis of the results described above, it was concluded that the active H^+^ efflux is functioning to maintain the intracellular pH constant against passive H^+^ influx according to the steep pH gradient across the cell membrane. The gene encoding the plasma membrane H^+^‐ATPase which is functioning in this active H^+^ efflux was cloned and sequenced (Ohta et al., 1997)l.

The enormous proton gradient across the cell membrane can be used for uptake of nutrients by effective H^+^‐symport (Gross, 2000). As the pH gradient decreases, the uptake of nutrients should be suppressed. Since the intracellular pH in *Cyanidium* cells is maintained at a constant within pH 6.8–7.0 regardless of external pH (Enami et al., 1986), the pH gradient across the cell membrane can be changed by the external pH. Thus, this study started from examination of the upper limit of acidity for *Cyanidium* survival and showed that it was pH 6. Furthermore, when the alga was cultured at the upper limit of acidity, the algal cells were found to survive under the stage of endospores and to secrete lysyl oxidase‐like protein (*Cyanidium* LOXL) as an extracellular enzyme.

Lysyl oxidases (LOX) and a number of closely related lysyl oxidase‐like proteins (LOXL) are widely distributed in the animal kingdom but have not been yet found in the plant kingdom (Csiszar, 2001; Kagan & Li, 2003; Lucero & Kagan, 2006). These proteins play a critical role in the formation and repair of the extracellular matrix by oxidizing lysine residues in elastin and collagen, thereby initiating the formation of covalent crosslinkages which stabilize these fibrous proteins (Kagan & Li, 2003). These proteins have been also revealed to exhibit a number of biological functions such as developmental regulation, tumor suppression, cell mobility, and cellular senescence, suggesting that these play exceptionally important roles beyond the induction of crosslinkage formation in collagen and elastin (Csiszar, 2001; Kagan & Li, 2003). Recently, Grau‐Bové, Ruiz‐Trillo, and Rodriguez‐Pascual (2015) have surveyed a wide selection of genomes in order to infer the evolutionary history of LOX(L) and found LOX(L) genomes not only in animals, but also in bacteria and archaea.

## 2 RESULTS

### 2.1 Upper limit of acidity for *Cyanidium* survival

**Figure 1:**
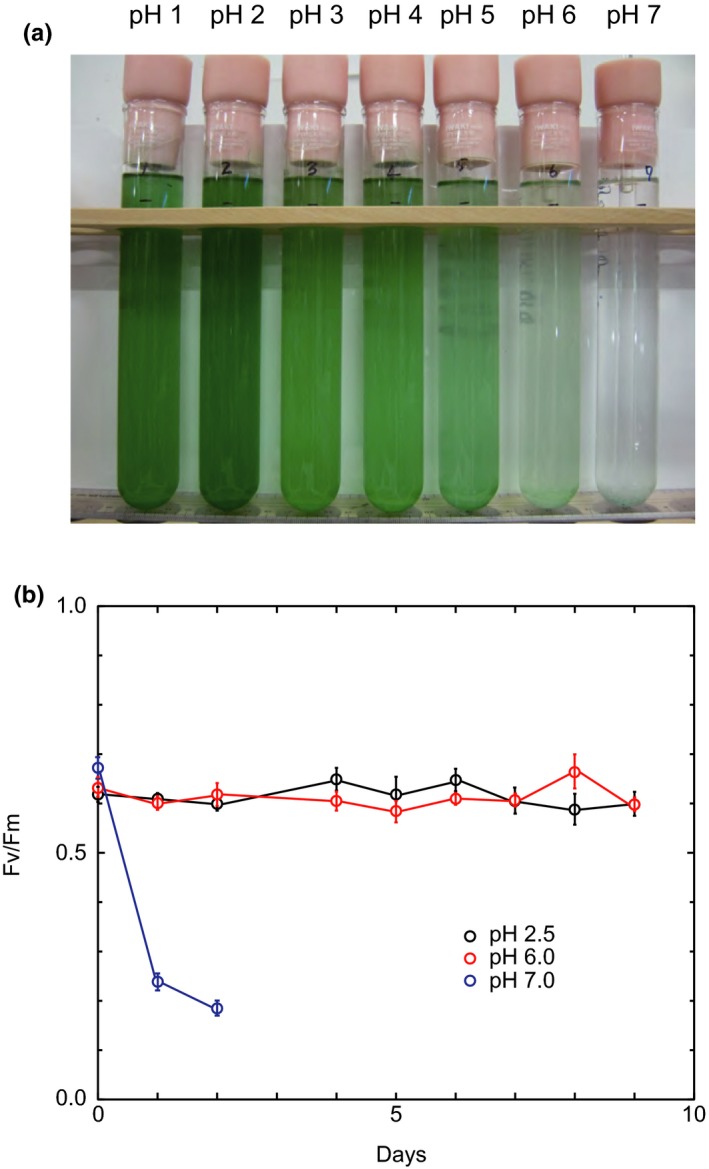
Photographs of *Cyanidium caldarium* which was cultured at various pHs (1–7) for 10 days (a), and Fv/Fm of intact cells of the alga cultured at pH 2.5 (black circles), at pH 6 (red circles), and at pH 7 (blue circles) for 0–9 days (b). Each value was obtained by repeating the experiment three times independently. Vertical bars show standard deviation

*Cyanidium caldarium* was cultured at a wide pH range (pH 1–7) to determine the upper limit of acidity for its survival. Figure 1a shows photographs of algal cells cultured at various pHs for 10 days. The cells grew optimally at pH 2 and grew well at a wide pH range of pH 1–5. In contrast, when the alga was cultured at pH 6, its original blue‐green color was kept but its color did not deepen, suggesting no multiplication of algal cells. Furthermore, the algal cells cultured at pH 7 showed complete chlorosis and died. This indicates that the upper limit of acidity is pH 6. To confirm this result, we measured induction of chlorophyll fluorescence from photosystem II (PS II) in intact cells cultured for 1–9 days at pH 2.5, 6, and 7. The *Fv* (*Fm* − *F*_*0*_)/*Fm* ratio, which is an estimate of the photochemical efficiency of PS II (Clarke, Hurry, Gustafsson, & Oquist, 1993; Herbert, Samson, Fork, & Laudenbach, 1992; Kvíderová, 2012), showed a constant value of about 0.6 in the cells cultured at both pH 2.5 and pH 6 even after 9 days (Figure 1b). This result indicates that the algal cells cultured at pH 6 retain the same photosynthetic activity as those cultured at pH 2.5. On the other hand, the *Fv*/*Fm* ratio of the cells at pH 7 declined to 0.185 after 2 days (Figure 1b), indicating that the alga lost the photosynthetic activity. Because the pH gradient across the cell membrane is nearly zero at external pH 7, the uptake of nutrients due to the pH gradient is completely suppressed, which may result in the inactivation of photosynthesis.

### 2.2 Suppression of cell division of *Cyanidium* cells at pH 6

**Figure 2:**
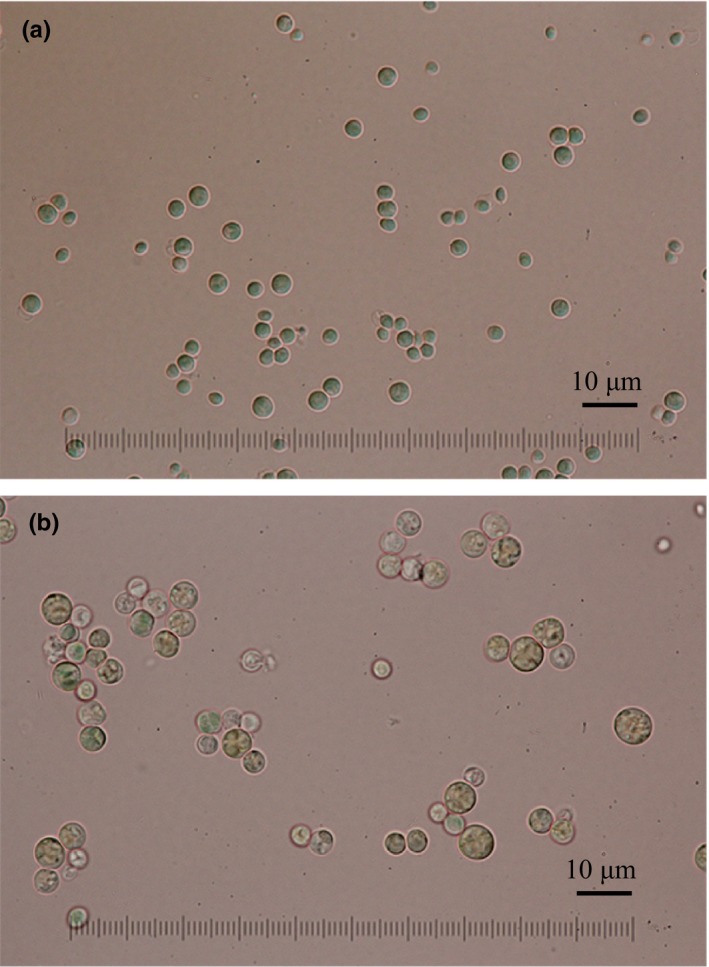
Micrographs of *Cyanidium caldarium* cultured at pH 2.5 (a) and at pH 6 (b). *Cyanidium* cells grown at pH 2.5 were subcultured in the medium at pH 6 and then cultured for 15 days

When *Cyanidium* cells were cultured at the upper limit of acidity (pH 6), microscopic observation showed that the number of large cells with four endospores increased significantly. As shown in Figure 2, most of the cells were small spheres of approximately 2–3 μm in diameter when cultured at pH 2.5 (a), whereas most cells became larger with four endospores of approximately 6–8 μm in diameter after 15 days of the culture at pH 6 (b).

**Figure 3:**
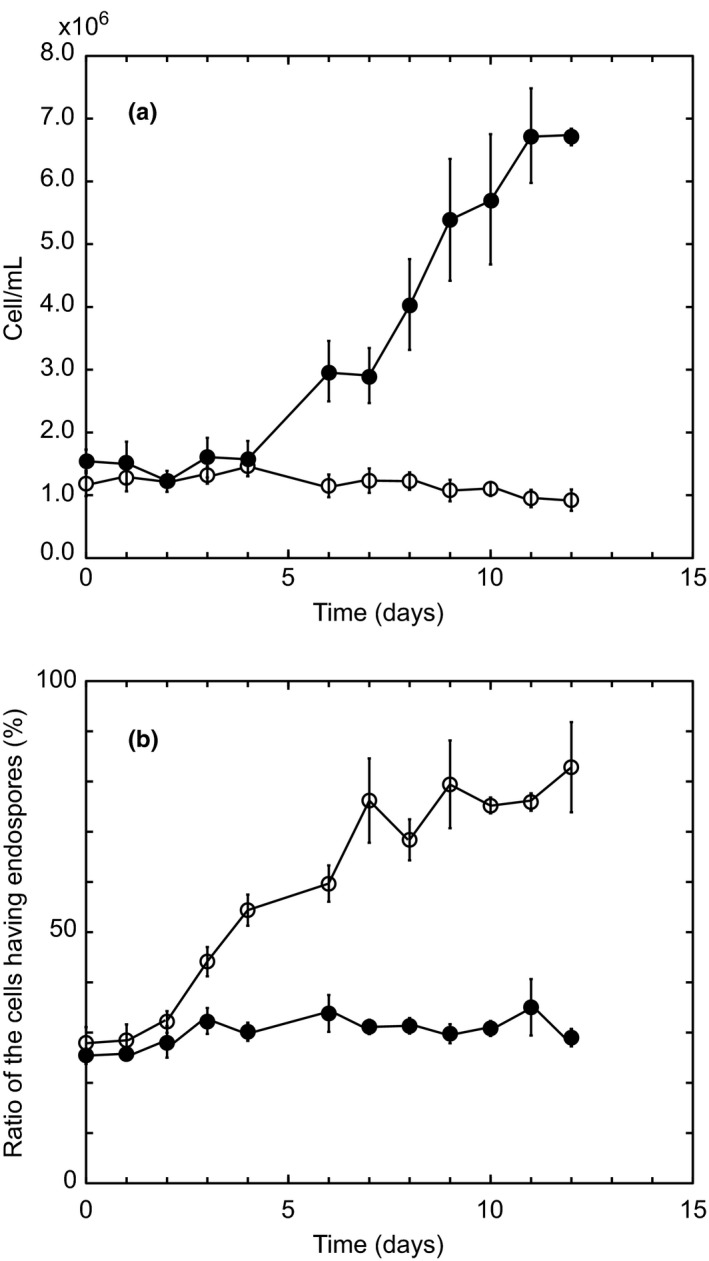
Growth curves (a) and ratio (%) of the cells with four endospores (b) of *Cyanidium caldarium* which was cultured at pH 2.5 (closed circles) and pH 6 (open circles). Each value was obtained by counting at least 500 cells and by repeating third times independently. Vertical bars show standard deviation

The number of cells and ratio of cells with four endospores were compared between algal cells cultured at pH 2.5 and those cultured at pH 6. Figure 3a shows the growth curves assayed by counting the cell number. When the alga was cultured at pH 2.5, the cell number logarithmically increased after lag periods of 4 days, while no increase in the number of cells was observed in the alga cultured at pH 6 even after 12 days. In Figure 3b, the ratio of cells with four endospores was compared between algal cells cultured at pH 2.5 and those cultured at pH 6. The ratio of cells with four endospores in algal cells cultured at pH 2.5 showed a constant value of 25%–30% through 1–12 days, whereas those in algal cells cultured at pH 6 significantly increased and reached approximately 80% after more than 7 days. These results indicate that *Cyanidium* cells cultured at pH 6 are able to form four endospores but not to split into daughter cells. This suggests that the alga survives in the endospores state at pH 6 and suppresses the increase in the number of cells to protect against nutrient uptake deficiently due to low pH gradient across the cell membrane.

**Figure 4:**
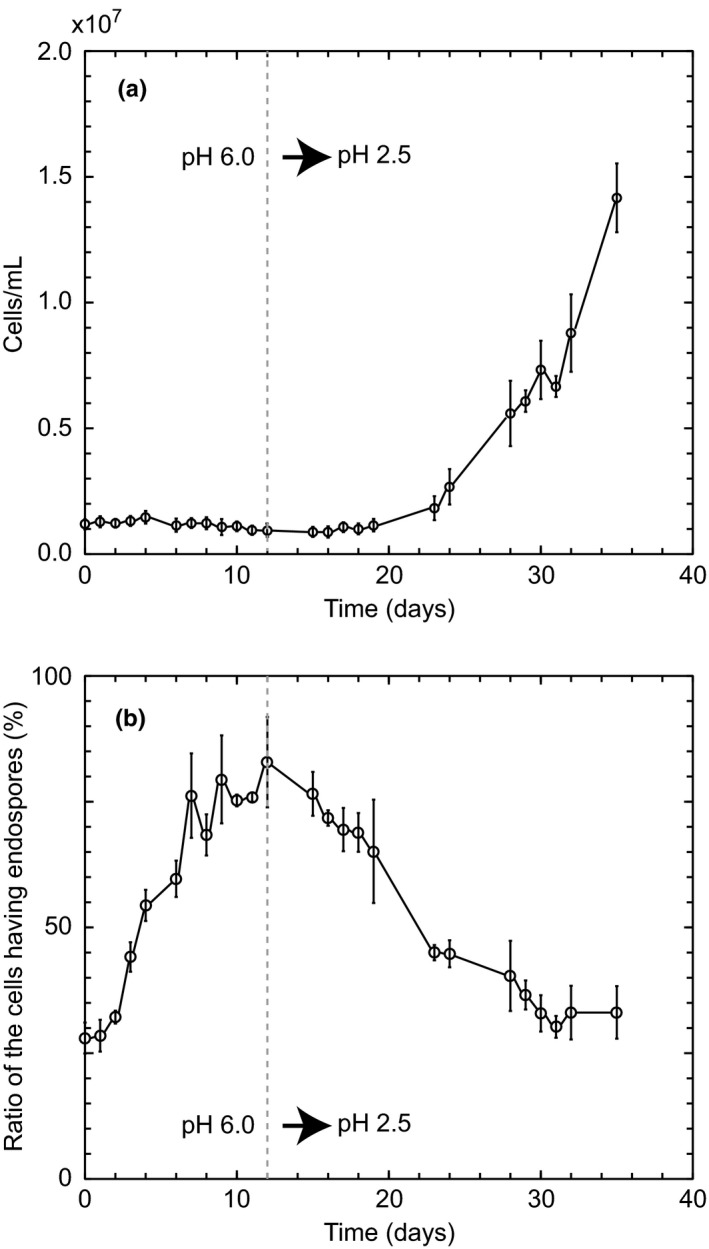
Growth curves (a) and ratio (%) of cells with four endospores (b) of *Cyanidium caldarium* which was cultured at pH 2.5 after the cultivation at pH 6 for 11 days. *Cyanidium* cells were cultured at pH 6 for 11 days and then centrifuged to remove medium at pH 6. Centrifuged precipitates were suspended in medium at pH 2.5 and then cultured. Each value was obtained by counting at least 500 cells and by repeating the experiment three times independently. Vertical bars show standard deviation

To confirm this suggestion, the algal cells cultured at pH 6 for 11 days were again cultured at pH 2.5. As shown in Figure 4a, the number of cells sharply increased after a long lag time of 10 days from changing to pH 2.5. The ratio of cells with four endospores gradually decreased and reached to about 30% after 25 days from changing to pH 2.5 (Figure 4b). This indicates that four endospores formed at pH 6 split into daughter cells at pH 2.5 in which sufficient nutrients are present due to the steep pH gradient across the cell membrane.

### 2.3 Secretion of an extracellular enzyme, lysyl oxidase‐like protein, from *Cyanidium* cells cultured at pH 6

**Figure 5:**
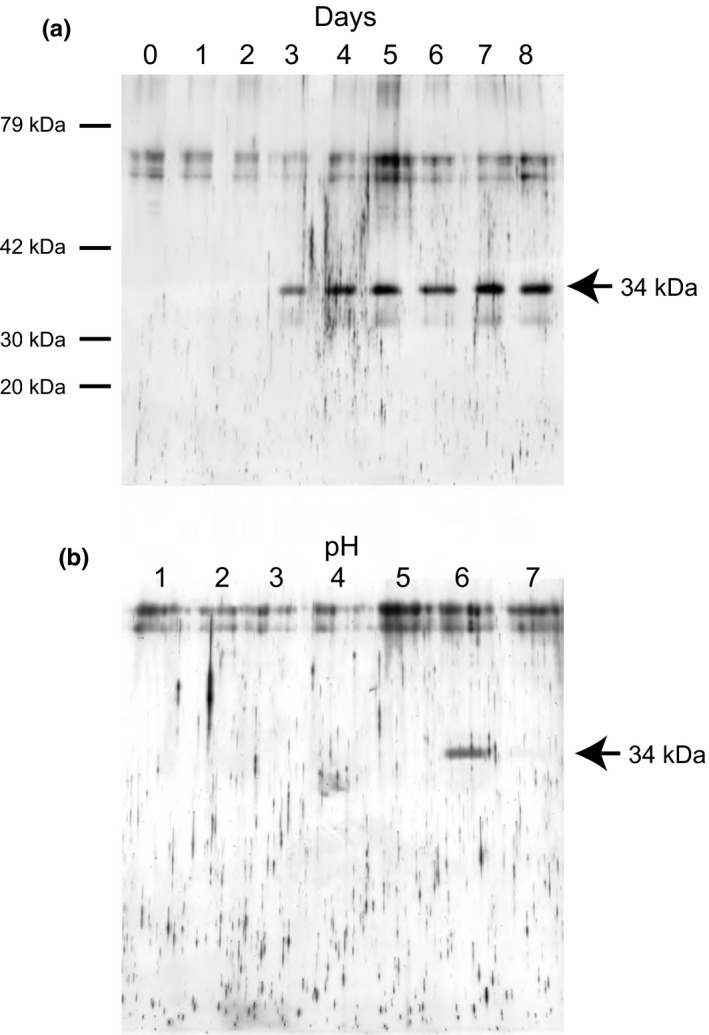
SDS‐PAGE profiles of extracellular protein secreted from *Cyanidium caldarium*. (a) Extracellular protein secreted from *C. caldarium* cultured at pH 6. The algal cells were cultured at pH 6 for 0–8 days. (b) Extracellular protein secreted from *C. caldarium* cultured at various pHs. The algal cells were cultured at various pHs for 7 days. Each sample was centrifuged at 5,000 × *g* for 5 min, and then 20 μl of the supernatants was applied to SDS‐PAGE

When *Cyanidium* cells were cultured at the upper limit of acidity (pH 6), we found that the culture medium foamed up remarkably, suggesting that some extracellular proteins are secreted from algal cells. To confirm this, peptide components of the centrifuged supernatants after precipitation of algal cells were analyzed by SDS‐PAGE. As shown in Figure 5a, one clear protein band appeared at an apparent molecular weight of 34 kDa after cultivation for more than 3 days.

The 34 kDa extracellular protein was secreted from algal cells cultured at pH 6 but was not secreted from those cultured at pH 1–5 and pH 7 (Figure 5b). In this study, the proteins secreted in the medium were examined but the proteins inside the cells or attached to cells were not examined. Thus, there are still two possibilities. (a) The 34 kDa extracellular protein is specifically expressed at pH 6. (b) The protein is expressed at all pHs but secreted in the medium specifically only at pH 6.

**Figure 6:**
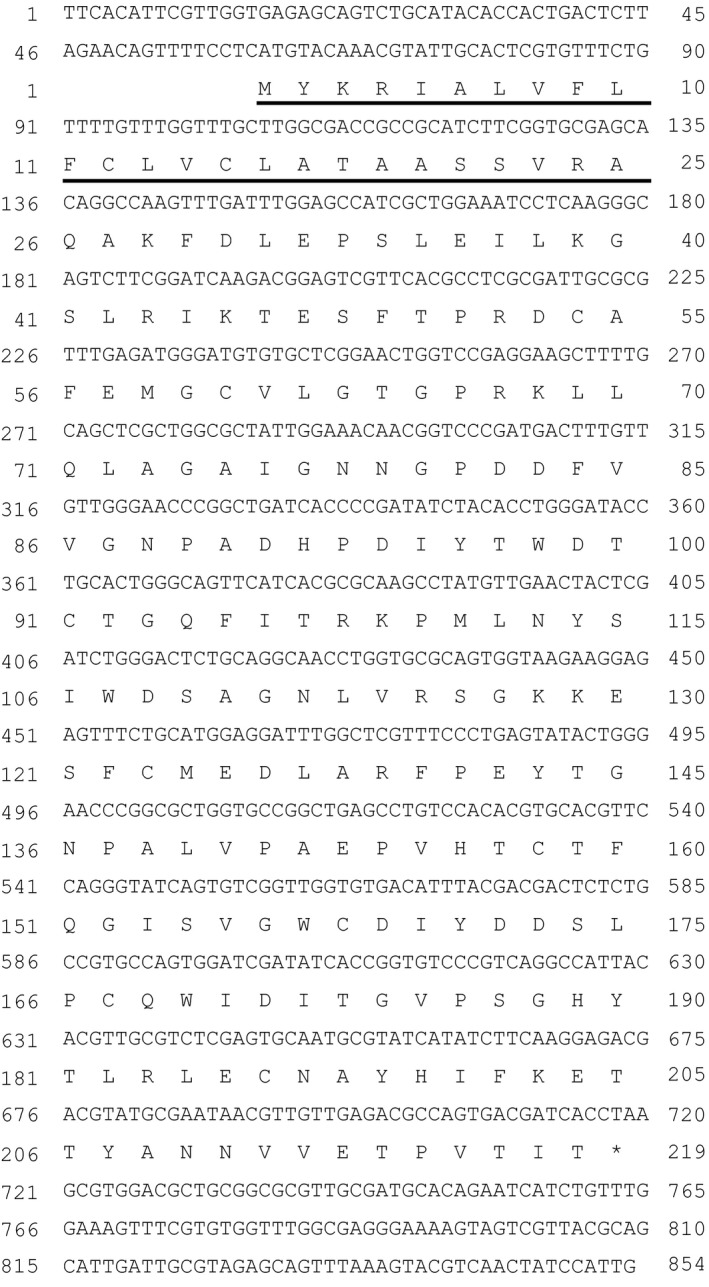
Nucleotide sequence of the gene encoding the 34 kDa extracellular protein secreted from *Cyanidium caldarium* cultured at pH 6. The deduced amino acid sequence is shown below the nucleotide sequence in the single‐letter code. The putative N‐terminal signal peptide (1–25; the underlined amino acid) was predicted using SOSUIsignal (Gomi et al., 2000, 2004)

**Figure 7:**
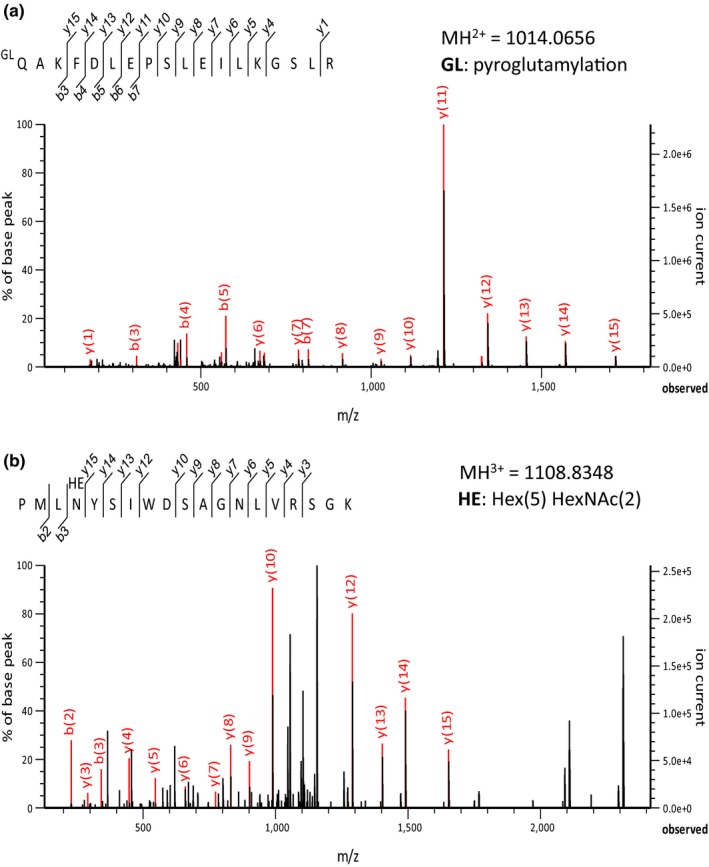
The MS/MS spectra corresponding to N‐terminal peptide (26–39) with pyroglutamate (a) and the peptide (110–128) with N‐linked high‐mannose oligosaccharides (b). The identified peptides were satisfied the Expectation value of <0.05 in MASCOT Database search

**Figure 8:**
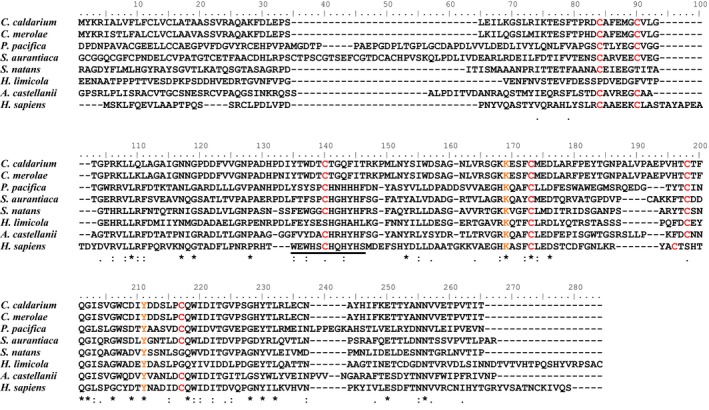
Alignment of the sequence of lysyl oxidase‐like protein from *Cyanidium caldarium*, with that of homologous proteins from other acidophilic alga (*Cyanidioschyzon merola*), myxobacteria (*Plesiocystis pacifica* and *Stigmatelia aurantiaca*), beta‐proteobacteria (*Sphaerotilus natans*)*,* halophilic archaea (*Haloterrigena limicola*), acanthamoeba (*Acanthamoeba castellanii*), and mammalian (*Homo sapiens*). The conserved “K” and “Y,” and “C” were highlighted in orange and red, respectively. The amino acid sequence corresponding to the copper‐binding site was marked by underline

The gene encoding the 34 kDa extracellular protein was successfully cloned according to the methods described in Methods section and its result is shown in Figure 6. This gene encodes a polypeptide of 219 amino acid residues with a total molecular mass of 24,217 Da. Its N‐terminal sequence was blocked by a possible modification, which was confirmed as pyroglutamylation (pyroglutamate formation at the N‐terminal located glutamine) using LC‐MS/MS analysis (Figure 7a). The putative N‐terminal signal peptide (1–25 corresponding to the underlined amino acids in Figure 6) was predicted using SOSUI‐signal (Gomi, Akazawa, & Mitaku, 2000; Gomi, Sonoyama, & Mitaku, 2004). These first 25 residues are considered to serve as a transit peptide for transferring the protein across the cell membrane. After removal of the signal peptide by cleavage at the Ala25–Gln26 position, the mature protein comprised 194 amino acid residues with a total molecular mass of 21,488 Da. Its sequence highly corresponded with that of LOXL protein from *C. merolae* CMN144C (Figure 8). Thus, the 34 kDa extracellular protein secreted from *Cyanidium* cells cultured at pH 6 was identified to be a lysyl oxidase‐like protein (LOXL).

MS/MS analysis of *Achromobacter* protease I digests of the *Cyanidium* LOXL showed that high‐mannose oligosaccharides were linked with the amide nitrogen of 113Asn in the peptide fragment of PMLNYSIWDSAGNLVRSGK in which Asn‐X‐Ser consensus sequence for N‐linked glycosylation was present (Figure 7b). Discrepancy between the apparent molecular mass by SDS‐PAGE (34 kDa) and the molecular mass calculated based on its amino acid sequence (21,488 Da) may, in part, be due to the N‐linked high‐mannose chains at 113Asn and may also be due to the presence of the cross‐linked cofactor (lysyl tyrosyl quinine [LTQ]: described after) which would prevent the complete unfolding of the mature protein under the conditions of SDS‐PAGE.

Lysyl oxidases encoding gene and LOX homologous genes were found to be widely distributed in the animal kingdom containing mammalian, bird, amphibian, fish, protochordate, echinoderm, arthropod, and hydra, as observed by basic local alignment search tool (BLAST) analysis using the DDBJ/EMBL/GenBank database (Table 1). In the plant kingdom, however, homologous genes were identified only in two acidophilic primitive red algae, *Cyanidium* and *Cyanidioschyzon*, but not in cyanobacteria, red algae, euglena, green algae, brown algae, diatoms, and all of higher plants. In bacteria, LOX homologous genes were found in myxobacteria (delta‐proteobacteria) and beta‐proteobacteria (Table 1) but not in alpha‐proteobacteria which is considered to be the origin of mitochondria (Esser et al., 2004; Gray, Lang, & Burger, 2004). Furthermore, in archaea, homologous genes were found only in two halophilic archaeon (*Haloterrigena limicola* and *Haloterrigena turknenica*) but not the other archaea (Table 1). The genes were also found in *Acanthamoeba castellanii*. As shown in Table 1, sequence identities between the mature protein (C‐domain) of *Cyanidium* LOXL and homologous proteins from the other organisms are relatively high: 97% with *Cyanidioschyzon*, 35%–38% with proteobacteria, and 29%–30% with mammalian LOX. These indicate that the C‐terminal domain of *Cyanidium* LOXL has been conserved through the species, although the N‐terminal regions involving signal sequences have been reported to be largely different among organisms (Csiszar, 2001; Kagan & Li, 2003; Lucero & Kagan, 2006).

**Table 1:** Comparison of the sequence identities and e‐values of lysyl oxidase and its homologous proteins between *Cyanidium caldarium* and various organisms

	Species	Identity (%)	e‐Value
Proteobacteria	*Sorangium cellulosum* So157‐2	38	3e‐32
	*Rhodoferax* sp. DCY110	35	8e‐29
	*Stigmatelia aurantiaca* DVV4/3‐1	38	7e‐28
	*Plesiocystis pacifica*	35	8e‐26
Archaea	*Haloterrigena limicola*	32	1e‐20
Acanthamoeba	*Acanthamoeba castellanii*	37	3e‐29
Red algae	*Cyanidioschyzon merolae*	97	9e‐142
Hydra	*Hydra vulgaris (magnipapillata)*	29	7e‐16
Arthropod	*Culex quinquefasciatus*	28	8e‐18
	*Acyrthosiphon pisum*	29	3e‐15
Echinoderm	*Strongylocentrotus purpuratus*	34	1e‐21
Protochordata	*Branchiostoma floridae*	32	8e‐22
	*Ciona intestinalis*	31	1e‐16
Fish	*Tetraodon nigroviridis*	31	7e‐16
	*Salmo salar*	29	2e‐17
	*Oncorhynchus mykiss*	31	5e‐17
Amphibian	*Xenopus laevis*	30	2e‐14
	*Xenopus tropicalis*	30	5e‐15
Bird	*Gallus gallus*	28	2e‐15
	*Taeniopygia guttata*	29	2e‐14
Mammalian	*Ornithorhynchus anatinus*	30	2e‐16
	*Mus musculus*	29	5e‐18
	*Bos tauru*s	29	1e‐19
	*Homo sapien*	29	5e‐17

*Note.* The sequence identities and e‐values were obtained based on ClustaIW (Larkin et al., 2007) and BLAST. The sequence identities (%) are expressed the amino acid sequence of mature part of *Cyanidium* lysyl oxidase‐like protein as 100%.

Figure 8 compared the sequence of *Cyanidium* LOXL with that of other organism LOX(L). The presence of LTQ, a unique peptidyl quinine functioning as a carbonyl cofactor, has been reported in human and rat LOX (Csiszar, 2001; Kagan & Li, 2003; Lucero & Kagan, 2006). The two amino acids, “K” and “Y,” involved in LTQ formation, have been completely conserved in acidophilic red algae (*C. caldarium* and *C. merolae*), myxobacteria (*Plesiocystis pacifica* and *Stigmatelia aurantiaca*), beta‐proteobacteria (*Sphaerotilus natans*)*,* halophilic archaea (*H. limicola*), and acanthamoeba (*A. castellanii*), indicating the presence of LTQ in LOXL of these organisms. Ten cysteines, which form five disulfide bonds and then contribute to the protein stability, have been also reported in animal LOX (Csiszar, 2001; Kagan & Li, 2003; Lucero & Kagan, 2006). Six in these 10 cysteines were conserved in the acidophilic algae, myxobacteria, and acanthamoeba LOXL, while only three or two cysteines were conserved in beta‐proteobacteria or archaea LOXL, respectively. Four histidine residues in amino acid sequences corresponding to the copper‐binding site (WXWHXCHXHYH) in human and rat LOX (Csiszar, 2001; Kagan & Li, 2003; Lucero & Kagan, 2006) were conserved in myxobacteria, beta‐proteobacteria*,* halophilic archaea, and acanthamoeba LOXL except for the first histidine, but were not conserved in acidophilic red algal LOXL, suggesting that acidophilic red algal LOXL may lack the copper‐binding site.

## 3 DISCUSSION

As sown in Figure 5a, *Cyanidium* LOXL secretion was detected after 3 days of the cultivation at pH 6. The large cells with four endospores also became to increase gradually after 3 days of the cultivation at pH 6 (Figure 3b). Thus, the large cell formation seems to be relevant to LOXL secretion. LOX in human and rat has been reported to function as the extracellular catalysis of lysine‐derived cross‐links in fibrillar collagens and elastin, playing a role in the formation and stabilization of extracellular matrix (Csiszar, 2001; Kagan & Li, 2003; Lucero & Kagan, 2006). *Cyanidium* cells have been also reported to have a thick cell wall, which is largely (50%–55%) proteinaceous (Bailey & Staehelin, 1968). Thus, it is likely that some proteins in the cell wall are cross‐linked with LOXL which results in stabilization of the cell wall, thereby preventing the endospores from splitting.

Another acidophilic primitive red alga, *C. merolae*, has also a similar LOXL gene. However, *C. merolae* are considered not to have the survival mechanisms similar to *Cyanidium* cells, because *C. merolae* lacks a rigid proteinaceous cell wall and reproduces by binary fission without formation of endospore (Matsuzaki et al., 2004; Miyagishima et al., 2001).

In this study, to the best of our knowledge, *Cyanidium* LOXL protein was identified for the first time in the plant kingdom. It should be noted that the *Cyanidium* LOXL protein consists of a simple amino acid sequence just of a highly conserved C‐terminal domain with only 25 residues of signal peptide, whereas mammal LOX and LOXL contain long N‐terminal sequences (about 200–500 residues) in addition to a highly conserved C‐terminal sequence (Csiszar, 2001; Kagan & Li, 2003; Lucero & Kagan, 2006). The diversifications in various mammal LOX and LOXL are considered to have been constructed during evolution of LOX by addition of various amino acids, such as signal peptides, proline‐rich region, and scavenger receptor cysteine‐rich domain to N‐terminal sequence regions (Csiszar, 2001; Kagan & Li, 2003; Lucero & Kagan, 2006). Thus, it is likely that the *Cyanidium* LOXL may be one of the origins of mammalian LOX.

In the plant kingdom, the LOX homologous genes are present only in two acido‐ and thermo‐philic primitive red algae, *Cyanidium* and *Cyanidioschyzon*. The genes had been lost in the third acido‐ and thermo‐philic red alga, *G. sulphuraria* which shows advance features and is closer to the lower *Rhodophyta* (Seckbach, 1987), and then completely disappeared in the plant kingdom. These facts suggest that *Cyanidioschyzon* and *Cyanidium* may be the first eukaryotic algae.

## 4 METHODS

### 4.1 Cultures

*Cyanidium caldarium* strain RK‐1 was cultured at 40°C under continuous illumination at 30 μmol/m^2^/s in an inorganic medium (2.64 g (NH_4_)_2_SO_4_, 0.544 g KH_2_PO_4_, 0.49 g MgSO_4_·7 H_2_O, 0.0148 g CaCl_2_·2H_2_O/L, and traces of microelements involving Fe, Mn, Co, Mo, Cu, EDTA). The pH was adjusted to each objective pH with the addition of 0.1 M H_2_SO_4_ or 1 M NaOH. During growth studies, the pH was checked at frequent intervals, and where necessary, 0.1 M H_2_SO_4_ or 1 M NaOH was added to bring the pH back to the initial values, and pH 1–3 were maintained within ±0.05 unit, and pH 4–7 within ±0.2 unit. *Cyanidium* cells cultured at pH 2.5 were used for subcultures in all experiments.

### 4.2 Measurements of chlorophyll fluorescence

The Chl fluorescence yield using single turn over flash analysis was measured by a double modulation fluorometer (Photon Systems Instruments). Cells were concentrated by low‐speed centrifugation and resuspended in fresh medium at a Chl *a* concentration of 5 μg/ml. The cell suspension was incubated in the dark for 10 min prior to the measurements.

### 4.3 Measurements of cell number and ratio of the cells with four endospores

*Cyanidium* cells cultured at pH 2.5 and pH 6 were observed under a differential interference contrast microscope, and number of cells and ratio of the cells with four endospores were counted using hemocytometer. Because cells cultured at pH 6 were significantly aggregated, the cells were shaken for 30 min in culture medium containing 0.01% Triton X‐100 to separate the aggregated cells before the microscope observation.

### 4.4 SDS‐PAGE

Samples were solubilized with 2.5% SDS and 3% 2‐merkaptoethanol for 10 min at room temperature. Solubilized samples were applied to a gel containing 12% acrylamide and 6 M urea. After electrophoresis at a constant current of 5 mA for 17 hr, gels were stained with a silver reagent and photographed.

### 4.5 Cloning and sequencing of the gene encoding the 34 kDa extracellular protein secreted from *Cyanidium caldarium*

Total RNA of the alga was isolated using Isogen reagent (Nippon Gene, Tokyo, Japan) by treatments with glass beads. Subsequently, full‐length cDNA appended with 5′ and 3′ adapter sequences was synthesized by RNA ligase‐mediated rapid amplification of cDNA ends (GeneRacer kit; Invitrogen, Carlsbad, CA). For the first PCR amplifications, primers were designed based on the sequences that are conserved among the LC‐MS/MS results and the sequence of LOXL protein from *C. merolae* CMN144C, which corresponded to partial sequences of the LC‐MS/MS results of the 34 kDa extracellular protein from *C. caldarium*. In the second PCR, sets of primer pairs were designed on the basis of sequence information obtained from the first PCR product and adapter sequences. Resultant sequences were combined to obtain the full‐length sequence of the cDNA. To confirm the gene sequence corresponding to the 34 kDa extracellular protein, the gene was amplified using specific primers from the 5′‐ and 3′‐untranslated regions. The PCR products obtained were inserted into the plasmid pCR2.1‐TOPO (Invitrogen, Carlsbad, CA), and the DNA sequences were determined using an ABI 3130 genetic analyzer (Applied Biosystems, Foster City, CA). At least three different clones were analyzed for each PCR product. Resulting sequences have been deposited at DDBJ/EMBL/GenBank under accession number LC314427.

### 4.6 Mass spectrometry

The silver‐stained protein band was cut out and de‐stained. The protein band was reduced with DTT (Wako Pure Chemical Industries), followed by carboxymethylation with iodoacetate (Sigma‐Aldrich). The band was digested with TPCK‐treated trypsin (Worthington Biochemical Corporation) or Achromobacter protease I (API; a gift from Takeharu Masaki, University of Ibaraki, Ibaraki, Japan) in 10 mM Tris (pH 8.0) at 37°C for 12 hr. The resulting peptide solution was separated on a nanoflow LC (Easy nLC 1000; Thermo Fisher Scientific) with a spray column (NTCC analytical column, C18, φ75 μm × 100 mm, 3 μm; Nikkyo Technos, Tokyo, Japan) with a linear gradient of 0%–35% buffer B (100% acetonitrile and 0.1% formic acid) at a flow rate of 300 nl/min over 10 min. The eluent was analyzed by nano LC‐MS/MS using a Q Exactive™ Hybrid Quadrupole‐Orbitrap Mass Spectrometer (Thermo Fisher Scientific) in a data‐dependent TOP 10 MS/MS method. The acquired data were searched against the in‐house database using an in‐house MASCOT server (version: 2.5; Matrix Science).

## AUTHOR CONTRIBUTIONS

T.T. and I.E. designed the research; A.O., T.S., O.M., R.K., N.I., and R.N. performed research; T.T., A.O., T.S., R.N., M.I., N.D., and I.E. analyzed data. T.T. and I.E. wrote the manuscript.
