# Supplementary material for: Lysyl oxidase‐like protein secreted from an acidophilic red alga, Cyanidium caldarium
Source: Plant Direct. 2018 Oct 8;2(10):e00084. doi: 10.1002/pld3.84 (PMC6508830; doi:10.1002/pld3.84)
Supplement: Supplementary file 1 [file PLD3-2-e00084-s001.pdf]

Answer to the reviewer 1

First of all, we are thankful to the reviewer for his/ her careful and thorough comments. After incorporation of the suggested revisions, we believe that the impact of this manuscript has been improved.

Reviewer comment 1: The following argument is still incomplete.
"When C. merolae was cultured at pH 6, the algal cells died and didn't secrete LOXL protein in the medium." If the authors merely observed cell death at pH 6, "and didn't secrete LOXL protein in the medium" should be deleted.

Answer: We agree with the reviewer's comment. We removed the sentence you suggested.
We revised the sentences as follows:

Another acidophilic primitive red alga, *Cyanidioschyzon merolae*, has also a similar LOXL gene. ~~When *C. merolae* was cultured at pH 6, the algal cells died and didn't secrete LOXL protein in the medium.~~ ~~Because *C. merolae* lacks a rigid proteinaceous cell wall and reproduces by binary fission without formation of endospore (Miyagishima et al., 2001; Matsuzaki et al., 2004), *Cyanidioschyzon* cells are considered not to have the survival mechanisms similar to *Cyanidium* cells.~~ However, *C. merolae* are considered not to have the survival mechanisms similar to *Cyanidium* cells, because *C. merolae* lacks a rigid proteinaceous cell wall and reproduces by binary fission without formation of endospore (Miyagishima et al., 2001; Matsuzaki et al., 2004).
